# Characterization of Human Tear Fluid by Means of Surface-Enhanced Raman Spectroscopy †

**DOI:** 10.3390/s19051177

**Published:** 2019-03-07

**Authors:** Carlo Camerlingo, Mikhail Lisitskiy, Maria Lepore, Marianna Portaccio, Daniela Montorio, Salvatore Del Prete, Gilda Cennamo

**Affiliations:** 1 Consiglio Nazionale delle Ricerche, SPIN-CNR, Via Campi Flegrei 34, 80078 Pozzuoli, Italy; carlo.camerlingo@spin.cnr.it; 2 Dipartimento di Medicina Sperimentale, Università della Campania “L. Vanvitelli”, 80138 Naples, Italy; maria.lepore@unicampania.it (M.L.); marianna.portaccio@unicampania.it (M.P.); 3 Dipt. di Neuroscienze e Scienze Riproduttive ed Odontostomatologiche, Universitá di Napoli ’Federico II’, 80121 Naples, Italy; da.montorio@gmail.com; 4 CISME—Centro Interdipartimentale di Microscopia Elettronica, Universitá di Napoli ’Federico II’, 80100 Naples, Italy; saldelp@gmail.com; 5 Dipt. di Sanitá Pubblica, Universitá di Napoli ’Federico II’, 80131 Naples, Italy; xgilda@hotmail.com

**Keywords:** SERS, tear, biomedical sensors, lactoferrin, lysozyme

## Abstract

Tears are exceptionally rich sources of information on the health status of the eyes, as well as of whole body functionality, due to the presence of a large variety of salts and organic components whose concentration can be altered by pathologies, eye diseases and/or inflammatory processes. Surface enhanced Raman spectroscopy (SERS) provides a unique method for analyzing low concentrations of organic fluids such as tears. In this work, a home-made colloid of gold nanoparticles has been used for preparing glass substrates able to efficiently induce an SERS effect in fluid samples excited by a He–Ne laser (λ = 633 nm). The method has been preliminary tested on Rhodamine 6G aqueous solutions at different concentrations, proving the possibility to sense substance concentrations as low as few μM, i.e., of the order of the main tear organic components. A clear SERS response has been obtained for human tear samples, allowing an interesting insight into tear composition. In particular, aspartic acid and glutamic acid have been shown to be possible markers for two important human tear components, i.e., lactoferrin and lysozyme.

## 1. Introduction

Human tears represent an exceptionally rich source of information regarding the health status of eyes and, more generally, of whole body functionality. This is mainly due to the presence in tears of a large variety of salts and organic components (including proteins, lipids, metabolites, nucleic acids, and electrolytes) whose concentrations can be altered by pathologies, eye diseases and/or inflammatory processes [1,2]. An increasing attention is presently given to the analysis of this human body fluid. The small amounts of substance considered and the typical low concentration of organic compounds hamper the access to direct analysis by biochemical methods even if the improvement of technology is overcoming some of the main obstacles and the route to a wider and reliable use of tear diagnostics is in progress. In this framework, micro-Raman spectroscopy can have an important contribution. Vibrational spectroscopies have been shown to be extremely useful to analyze biological samples providing valuable insight into the nature of samples and biofluids [3,4,5,6] and into the determination of the chemical structure of specific molecules [7]. Raman spectroscopy has been also used for investigating tears [8,9,10]. More recently, the use of Surface Enhanced Raman Spectroscopy (SERS) has been proposed with interesting perspectives in terms of sensitivity and specificity of the signal response [11,12,13]. In the work of P. Hu et al. [11] SERS has been implemented by using silver nanoparticles and applied to the analysis of dried human tears. The spectroscopy of dried fluids, the so-called drop-coating deposition Raman spectroscopy (DCDRS), has many advantages with respect to liquid sample analysis and has been applied fruitfully to tear investigation [8,9]. SERS mechanisms add new potentialities to DCDRS. In the case of tears, SERS response has some differences with respect to DCDRS signal and the observed modes are mainly assignable to the proteins [11]. In particular, P. Hu et al. [11] evidenced a correlation between the intensity of SERS modes at 757 and 1557 cm${}^{-1}$ spectral positions and the lysozyme content, in agreement with a previous work of J. Filik and N. Stones [14]. Gold coated nanostructured substrates have been used by S. Choi et al. to perform SERS investigation of dried tears aimed to characterize the spectral changes occurring in adenoviral conjunctivitis-infected subjects [12]. In that work, the principal component analysis (PCA) allowed a global analysis of the spectrum configuration that was able to distinguish between normal and infected tear fluids confirming that SERS measurements can offer valuable diagnostic tools. For this reason, we investigate the possibility of performing SERS measurements on human tears using a cheap and easy method to prepare home-made substrates, differently from reported works that used more sophisticated and expensive substrates [11,12] not suitable for large screening purposes. In this present work, human tear samples have been examinated by SERS using home-made SERS subtrates based on gold nanoparticles using a procedure simpler and faster than the one proposed in [11], which requires the mixing of each tear sample with colloids. In order to test the efficiency and sensibility of the method, preliminary SERS measurements have been performed on Rhodamine 6G aqueous solutions at different concentrations.

## 2. Materials and Methods

### 2.1. Tears Collection

Tear specimens have been collected from healthy volunteers. Informed consent has been preliminarly obtained from them. Smooth edge sterile capillary glass tubes (ACCU-FILL 90 MICROPET by Becton, Dickinson and Co, Clay Adams CA USA) with internal diameter = 0.2 mm, outside diameter = 1 mm and length = 12.7 mm have been used. During the collection, the volunteers’ lower eyelids were gently pulled down and the tip of the open capillary tube placed in contact with the tear meniscus without irritating the conjunctiva. A minimum of 1.0 μL tear was collected. The samples were stored at 4 ${}^{\circ }$C and tested by SERS within 7 days. In this work, eight healthy patients aged between 33 and 85 years have been considered. The list of patients, their birth year and sex is reported in Table 1.

### 2.2. SERS Implementation

SERS has been induced by using home-made Gold nanoparticles (GNP) onto conventional microscope slides. A colloid of GNPs has been obtained by a conventional citrate reduction method [15,16]. A 0.01% HAuCl${}_{4}$ solution was reduced by 1% sodium citrate with vigorous stirring at near boiling temperature. The amount of sodium citrate was defined in order to obtain GNPs with controlled size with particle diameter in the range of 20–50 nm. Preliminary tests have shown that the GNPs with a diameter equal to ∼27 nm give optimum SERS features [16].

Particle size, preparation stability and Raman signal of the resulting preparations have been investigated by means of absorption spectroscopy, Transmission Electron Microscop (TEM), and Dynamic Light Scattering (DLS). In particular, absorption spectroscopy was used to get information on the plasmon resonance peak and to study the stability of the preparations [17,18]. TEM and DLS were employed to estimate the size of the GNPs. Absorption spectra of the prepared GNPs were recorded by UV–VIS spectroscopy by a two-beams spectrophotometer (LS25, Perkin Elmer, Waltham, MA, USA).

Hydrogen tetrachloroaurate (HAuCl${}_{4}$), and trisodium citrate were purchased from Sigma-Aldrich (Sigma-Aldrich Co., St. Louis, MO, USA). All chemicals were used as received. Rhodamine 6G were purchased from Sigma-Aldrich (St. Louis, MO, USA).

### 2.3. SERS Measurements

A small amount of samples to be analyzed was dropped on the dried residual of the GNP colloid, and quickly measured. SERS measurements were performed by using a Jobin–Yvon system from Horiba Scientific ISA (Osaka, Japan), equipped with a TriAx 180 monochromator, a liquid nitrogen cooled charge-coupled detector and a 1800 grooves/mm grating (final spectral resolution: 4 cm${}^{-1}$). The spectra were recorded in air at room temperature using a 17 mW He–Ne laser source (wavelength 632.8 nm). Accumulation times in the 30–180 s range were used. The laser beam was focused to a 2-μm spot size on the sample through an Olympus microscope equipped with 100× optical objective. Raman spectroscopy measurements were also done for comparison, by using conventional microscope slides as substrates. Acquisition times and modalities were similar to those used for SERS measurements.

### 2.4. Data Analysis

Before being analyzed and compared, the spectra were numerically elaborated in order to eliminate background signal, limit the signal noise and normalize data. For this purpose, we used mathematical algorithms based on “wavelet” functions. Widely used in the signal analysis, “wavelets” provide an efficient basis for the hierarchical spectrum representation through the so called Discrete Wavelet transform (DWT) [19]. Biorthogonal wavelets based on the B-spline function were used in the framework of software package ‘wavelet toolbox’ of MATLAB program (by MathWorks Inc.). Low and high scale components of the signal DWT were not be included in the final reconstructed signal, taking away the background and part of the uncorrelated noise, respectively. Finally, the amplitude of the spectral data was normalized by using vector normalization, i.e., by scaling the spectral data set suitably to obtain the standard deviation with respect to the average value equal to 1. The spectra were analyzed in terms of convoluted lorentzian-shaped vibrational modes by performing a best-fit procedure based on the Levenberg–Marquardt nonlinear least-square methods.

## 3. Results

**Figure 1:**
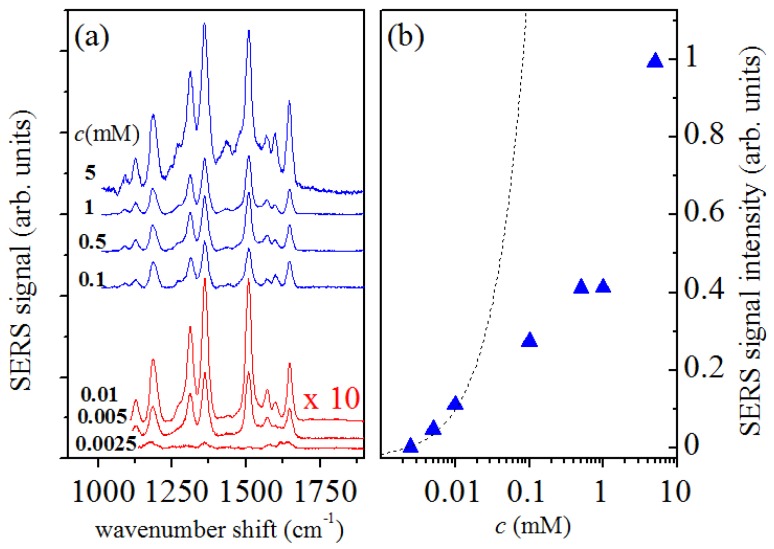
(**a**) SERS of Rhodamine aqueous solution at different concentration in the range of 0.0025–5 mM. The intensity of spectra reported in red are amplified by a factor 10. The spectra are reported arbitrarly shifted along the y-axis. (**b**) dependence of SERS signal intensity (SERS mode at 1504 cm${}^{-1}$) on the Rhodamine 6G concentration (log scale). The linear dependence is represented by the dotted line.

Some preliminary SERS measurements on Rhodamine 6G diluted in deionized water at different concentrations were performed in order to quantify the effect of the Raman response enhancement. SERS spectra were collected on the Rhodamine–water solutions in similar conditions, by using an acquisition time of 30 s. The obtained spectra are reported in Figure 1a, arbitrarily shifted along the y-axis in order to improve the readability. They refer to Rhodamine 6G solutions with concentrations of c= 0.0025, 0.005, 0.01, 0.1, 0.5, 1 and 5 mM, respectively. The intensities of the spectra referring to lower concentrations (red lines) are reported as being magnified ten times with respect to the remaining spectra (blue lines). The spectra observed are consistent with data reported in literature [20] and prove the high sensitivity of the method. The dependence of SERS signal intensity on the Rhodamine 6G concentration is shown in Figure 1b, where the intensity values of the spectral mode at 1504 cm${}^{-1}$ are reported as a function of the solution concentration *c*. The data have been obtained by fitting the Raman spectra of Figure 1 by Lorentzian functions. For low-concentration values the SERS signal intensity increases approximately linearly with concentration, as visible in Figure 1b where the linear dependence is represented by a dotted line. When concentration is larger than 10 μM SERS signal is lower than the value expected in the case of a linear dependence on *c*, indicating a saturation trend probably related to a filling effect of GNP surfaces involved in the SERS mechanism. A SERS efficiency of about four times $10^{3}$ has been estimated by comparing the SERS data of *c*=5μM solution with the signal obtained by conventional Raman spectroscopy on the *c*=5 mM solution.

**Figure 2:**
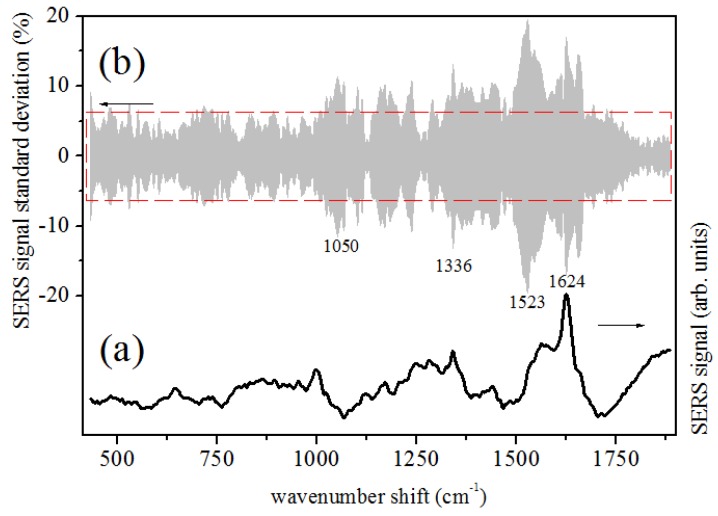
(**a**) Human tear SERS signal. (**b**) SERS signal standard deviation with respect to the average signal (bottom spectrum) of tears. The red box indicated the mean value of the signal standard deviation (6.3±3.3%).

**Figure 3:**
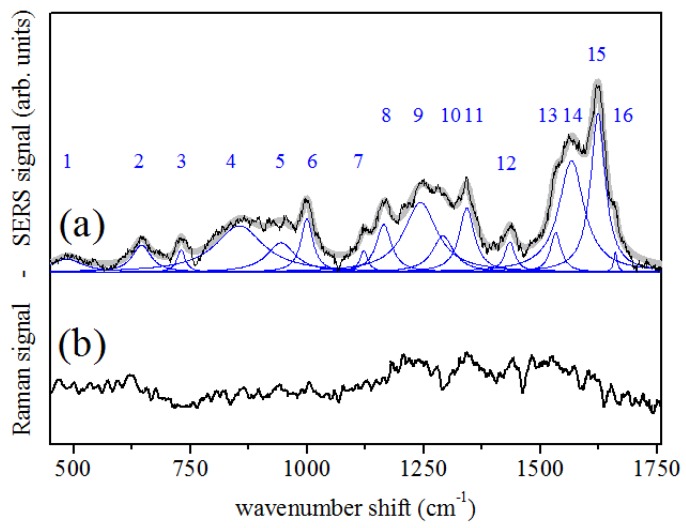
(**a**) SERS spectrum of human tears. The experimental data were fitted by a convolution of Lorentzian functions (numbered blue peaks) representing the main Raman modes occurring in the sample (see Table 2 for a temptative assignment of the Raman modes. (**b**) Conventional Raman spectrum of dried human tears (the signal in (**a**,**b**) are arbitrary scaled).

SERS signals from tears were collected in conditions similar to those used for the preliminary tests on Rhodamine 6G. In this case, the acquisition time was typically 180 s. Three or more acquisitions were performed for each sample, using different points of the sample area. The relative standard deviation of the SERS signals obtained from each sample was typically lower than 5%. The spectrum reported in Figure 2a is obtained by averaging SERS responses of tear samples obtained from the eight considered healthy patients. In order to quantify similarities or possible differences among the samples and the repeatability of the measurement, the standard deviation with respect to the average values was calculated for each point of the average SERS signal, and the variation range of SERS response, due to patients’ individual peculiarities, is reported in Figure 2b. A mean standard deviation σ=6.3±3.3% was calculated with respect to averaged signals (bottom spectrum of Figure 2). The signal dispersion is generally lower than 10% of the SERS signal, even if a larger signal deviation range occurs in some points of the spectra, at about 1050, 1336, 1523 and 1624 cm${}^{-1}$. Amide I and Amide III Raman bands are clearly seen in the reported spectrum at the wavenumber Raman-shifts of ∼1600 cm${}^{-1}$ and ∼1250 cm${}^{-1}$, respectively. In Figure 3a, the result of the spectrum deconvolution in terms of Lorentzian components (reported as blue lines) obtained by a numerical fitting procedure is reported. The main component modes are determined and listed in Table 2, with a temptative assignment of the vibrational sources, based on SERS data of proteins available in the literature [12,21,22]. The SERS signal of Figure 2a is compared with the conventional Raman spectrum measured on a human tear dried drop (Figure 2b). Acquisition times and modalities were similar to those used for SERS measurements, but a significant signal intensity increase and improvement in the spectral resolution are noticed in the SERS signal when the two spectra are compared. It is worth to note that the spectroscopy measurements here reported employed lower laser power and/or photon energy (i.e., a lower Raman scattering efficiency) with respect to those used in the works of Filik [14] and Hu [11] on dried tear drops. This evidences that the observed spectra are due to SERS mechanisms instead of a mere optimization of the Raman spectroscopy collection, as in the case of DCDRS experiments.

## 4. Discussion

The modes featured by the spectrum of tears reported in Figure 3 and listed in Table 2 are mainly concerning proteins and can be temptatively assigned to amino acids [22]. The main components occurring in tears are immunoglobulins (IgA), lactoferrin, lysozyme, lipocalin and albumine [10,23]. The molecular weights and the typical concentrations in human tears of these substances are listed in Table 3. The concentrations are of the order of few mg/mL [10], thus, compatible with SERS sensitivity estimated by Rhodamine 6G measurements, even if each substance could have a different response efficiency regard to SERS mechanisms.

Among the tear components, lactoferrin (LF) and lysozyme (LZ) have an important role for eye functionality providing defence mechanisms against infective agents [1]. A significant decrease of their levels has been reported in patients suffering from inflammatory Dry Eye Disease (DED) [24,25,26]. LF is an iron-binding protein present in almost all body biofluids. It has a proven anti-bacterial and anti-inflammatory ability [27]. The main components of the LF are Glutamic and Aspartic acids, Leucine, Arginine, Lysine, Valine and Phenylalanine [28]. LZ is an antimicrobial enzyme constituted by a single chain polypeptide. Its main components are Aspartic acid, Alanine, Glycine, Arginine, Serine, Leucin and threonine [29]. A direct correlation of SERS signal to the LF and LZ is not seen due to the complex and rich composition of tears. However, the peak assignments done in Table 2 for Glutamic acid (at 1243 cm${}^{-1}$ and 1434 cm${}^{-1}$) and for Aspartic acid (1342 cm${}^{-1}$) provide potential markers for LF and LZ, respectively. These modes are in agreement with Raman spectra reported for single-component solutions of LF and LZ [11,14] and with SERS response of LZ [30]. In particular, the SERS spectrum of LZ reported by Jun Hu et al. [30] exhibited a particularly intense peak at 1358 cm${}^{-1}$ stronger than the ones observed at 1280 and 1432 cm${}^{-1}$. Furthermore, modes at 730 cm${}^{-1}$ and 1567 cm${}^{-1}$ should be related to LZ, in agreement with Ref. [14,30]. In a recent SERS study on human tears, W.S. Kim et al. found a correlation between SERS signal intensity ratio at 1342 cm${}^{-1}$ and 1242 cm${}^{-1}$ and the infection state of eye [13]. The authors noticed an increase of the *I*${}_{1342}$/*I*${}_{1242}$ ratio value in patients affected by Adenovirus (*I*${}_{1342}$/*I*${}_{1242}$ = 1.13) or Herpes Simplex (*I*${}_{1342}$/*I*${}_{1242}$ = 3.73) diseases. These values are significatively higher than the *I*${}_{1342}$/*I*${}_{1242}$ = 0.8 estimated for reference healthy patients. This feature could be explained in terms of a decrease of the LF level originated by the infection state. LZ level should not change significantly because, in agreement with oculist phenomenology, patients with blepharitis, conjunctivitis, and keratitis had normal mean LZ content of tears while patients with herpes simplex keratitis had low LZ values [31]. In our case, we estimated an average value of *I*${}_{1342}$/*I*${}_{1242}$ = 0.9±0.3 for the considered height healthy patients.

## 5. Conclusions

The potentiality of SERS for characterizing tears has been investigated by using home-made fabricated Gold-nanoparticle-based substrates. The method has been previously tested and characterized on water diluted Rhodamine 6G samples. Human tear fluids from eight healthy patients have been considered. The SERS response results are mainly related to amino acids and provided a valuable source of information even if the interpretation is not immediate and more work should be done in order to have a more complete and exhaustive data comprehension. Nevertheless, assignments for two components of tears have been determined at the spectral positions of 1243 cm${}^{-1}$ and 1434 cm${}^{-1}$ (lactoferrin) and 1342 cm${}^{-1}$ (lysozyme). As widely reported in the literature and discussed above, the concentrations of both these components are affected by eye health state, and a change in SERS intensity is expected in the case of pathologies. A quantitative assessment of main tear components (in particular, Lysozyme and Lactoferrin) is undoubtedly a demanding issue and a challenge for the future progress of SERS as a diagnostic method. The promising results that are reported here allow us to estimate a future development of research activity towards the implementation of methods suitable to a widespread application of SERS methods in oculistic practice. The development of cheap and friendly methods for SERS implementation, as the one considered in this work, is a first step towards this aim. An interesting future perspective is constituted by the development of soft substrates for SERS implementation, properly designed by using paper or tissues embedded in metallic nanoparticles [13,32,33].

## Figures and Tables

**Table 1:** List of patients.

id. Number	Birth Year	Sex
A	1985	f
B	1980	f
C	1972	f
D	1956	f
E	1952	m
F	1948	m
G	1943	f
H	1933	m

**Table 2:** Principal contributions in SERS spectrum of human tears and their assignment according to Refs. [12,21,22].

nr	Center (cm${}^{-1}$)	Assignment	Component
1	484	Ring def.	amino acids
2	645	COO${}^{-}$ wag.	amino acids
3	730	COO${}^{-}$ def.	amino acids
4	855	C-C str.	amino acids
5	944	C-C str.	amino acids
6	1000	symm. ring CC str.	phenylalanine
7	1121	NH${}_{3}^{+}$ def.	amino acids
8	1165	N-H wag.	amino acids
9	1243	Amide III β-sheet, CH${}_{2}$ wag.	protein
10	1291	Amide III α-helix CH${}_{2}$ wag.	protein
11	1342	C-H def.	Aspartic acid, amino acids
12	1435	CO${}^{-}$ symm. str.	Glutamic acid, amino acids
13	1533	C-C str.	amino acids
14	1567	NH${}_{2}$ sciss.	amino acids
15	1624	Indole N-H, C=O str.	amino acids
16	1661	Amide I α-helix	protein

(def.: deformation; wag.: wagging; str.: stretching; sciss.:scissoring).

**Table 3:** Main components of human tears.

Component	Mol. Weight (KDa)	*c* (mg/mL) [10]	*c* (μM)	Description
lactoferrin	80	1.8–2.7	23–34	iron binding glycoprotein
lysozyme	14	1.6–2.5	111–172	single chain polypeptide
lipocaline	20	1.2–2.95	62–145	low mol. weight protein
immunoglobulins (IgA)	162	0.2–0.3	1.5–1.9	glycoproteins (antibodies)
albumine	66	1.3	20	single peptide chain
